# Combining MAD and CPAP as an effective strategy for treating patients with severe sleep apnea intolerant to high-pressure PAP and unresponsive to MAD

**DOI:** 10.1371/journal.pone.0187032

**Published:** 2017-10-26

**Authors:** Hsiang-Wen Liu, Yunn-Jy Chen, Yi-Chun Lai, Ching-Yi Huang, Ya-Ling Huang, Ming-Tzer Lin, Sung-Ying Han, Chi-Ling Chen, Chong-Jen Yu, Pei-Lin Lee

**Affiliations:** 1 Department of Internal Medicine, National Taiwan University Hospital, Yun-Lin Branch, Yun-Lin, Taiwan; 2 Graduate Institute of Epidemiology and Preventive Medicine, College of Public Health, Taipei, Taiwan; 3 Department of Dentistry, School of Dentistry, Graduate Institute of Clinical Dentistry, Taipei, Taiwan; 4 Department of Internal Medicine, National Yang-Ming University Hospital, I-Lan, Taiwan; 5 Department of Internal Medicine, National Taiwan University Hospital, Taipei, Taiwan; 6 Department of Internal Medicine, Hsiao Chung-Cheng Hospital, New Taipei, Taiwan; 7 Department of Dentistry, Mackay Memorial Hospital, Taipei, Taiwan; 8 Graduate Institute of Clinical Medicine, College of Medicine, National Taiwan University, Taipei, Taiwan; 9 Center of Sleep Disorder, National Taiwan University Hospital, Taipei, Taiwan; National Yang-Ming University, TAIWAN

## Abstract

**Introduction:**

This study aimed to determine the effect of combining positive airway pressure (PAP) therapy and mandibular advancement device (MAD) in patients with severe obstructive sleep apnea (OSA) who were pressure intolerant for PAP and were unresponsive to MAD.

**Methods:**

This retrospective study reviewed the medical records of severe OSA patients with apnea-hypopnea index (AHI) ≥ 30/hr who were diagnosed between October 1, 2008 and June 30, 2014. Patients were initially treated with 2 weeks of PAP, and those who were intolerant to high-pressure PAP (≥15 cm H_2_O) were switched to 12 weeks of MAD, which is a monobloc designed at 75% of maximum protrusion. Patients who had high residual AHI (≥15/hr) on MAD underwent 12 weeks of combination therapy (CT) with MAD and CPAP and were enrolled in the present study. Enrolled subjects who completed the 12-week CT were followed-up until June 30, 2016.

**Results:**

A total of 14 male patients were included. All three treatments effectively reduced AHI, oxygen desaturation index (ODI), and total sleep time with SpO_2_ <90% (% TST-SpO_2_<90%) compared to pretreatment values. The residual AHI and ODI on CT was lower than that on MAD or PAP. The residual % TST-SpO_2_<90% was lower than that on MAD and similar to that on PAP. The therapeutic pressure on CT was on average 9.2 cm H_2_O lower than that on PAP. For the 11 patients who completed CT, only CT reduced ESS compared to pretreatment value. No treatment had significant impact on % slow wave sleep or overnight change of blood pressure. For patients who completed CT, the average usage was 5.9±1.7 hr/night at 12th week and 6.4±1.5 hr/night at a median follow-up of 36.5-months.

**Conclusions:**

Combining MAD and CPAP showed additive effects on reducing AHI and ODI, and lowered the therapeutic pressures.

## 1. Introduction

Obstructive sleep apnea (OSA) is characterized by repeated collapse of the upper airways during sleep, resulting in chronic intermittent hypoxia and sleep fragmentation [1]. The standard therapy for patients with moderate-to-severe OSA is continuous positive airway pressure (CPAP), which can effectively improve daytime alertness, functional status, blood pressure (BP), and quality of life [2]. However, the beneficial effect of CPAP therapy often relies on patient compliance. The use of CPAP for ≥4 hours per night is considered good compliance in terms of lowering BP and improving subjective sleepiness, whereas ≥6 hr/night is needed to improve daytime function [3, 4]. The compliance with CPAP is often problematic; studies report rates of good compliance at 46–53% [5, 6]. Factors associated with poor compliance are low apnea-hypopnea index (AHI), non-sleepiness, nasopharyngeal discomfort, and lack of subjective effect [7–10]. Pressure intolerance is a common self-reported complaint; however, the impact of CPAP pressure on compliance has rarely been studied. Although there is only limited evidence that associates high pressure with poor compliance [11], the American Academy of Sleep Medicine (AASM) suggests transitioning CPAP to bilevel positive airway pressure (BPAP) when the therapeutic pressure of ≥15 cm H_2_O is required to eliminate apnea-hypopnea episodes [12].

Mandibular advancement device (MAD) is an alternative for patients with severe OSA who cannot tolerate CPAP [13, 14]. By advancing the mandible, MAD can dilate the pharyngeal aerospace, especially the lateral diameters [15–17]. Although MAD is less effective in reducing AHI compared to CPAP, it is as effective as CPAP in improving daytime alertness, neuro-psychologic function, quality of life, and BP [13, 18–21]. Such effects may be attributed to better tolerance and patient preference [19].

It is not well documented how combining MAD with CPAP may benefit OSA patients who are intolerant to high-pressure PAP and unresponsive to MAD. El-Solh *et al*. tested the effects of a 3-day combination therapy (CT) of CPAP and MAD at ≥65% maximum protrusion (MaxP) in 10 OSA patients who were intolerant of CPAP due to high pressure and had residual AHI ≥5/hr on MAD. The results showed that the CT reduced AHI and desaturation event as effectively as CPAP, while lowering the therapeutic pressure compared to CPAP alone [22]. However, OSA severity (AHI 6-55/hr) and the self-reported intolerable pressure (as low as 6 cm H_2_O) varied greatly among subjects. In addition, the study only lasted for 3 days; the long-term effect on compliance was unknown. Vries et al. reported the effect of a 12-week CT of CPAP and MAD (70% of MaxP) in 5 patients with moderate-severe OSA who could tolerate CPAP. Although the result showed no difference between CT and CPAP at residual AHI, the therapeutic pressure was lowered in all patients by an average of 5.1 cm H_2_O, and CT was preferred over CPAP.[23]

Kaminska et al. assessed the impact of MAD in the neutral position on therapeutic pressure in 6 moderate-severe OSA patients who required higher effective pressure (P_eff_) on oro-nasal mask than on nasal mask. Combing MAD with CPAP did not affect the Peff in all patients with nasal mask (10.4±3 vs 11.1±2.6 cmH_2_O), but reduced the oro-nasal P_eff_ in 3 subjects, including two who could not be treated with nasal mask (P_eff_ >20 cm H2O) [24]. The result suggests that MAD may have partially reversed the posterior mandibular displacement caused by oro-nasal mask. It is unknown if the therapeutic pressure would be further reduced with MAD at an advanced position.

Though the effect of CT is inconclusive, the preliminary success of CT reported by these studies warrant further investigation. The present study aimed to evaluate the therapeutic potential of combining MAD and CPAP in a well-defined cohort of severe OSA patients who were intolerant to high-pressure PAP of ≥15 cm H_2_O and had inadequate response to MAD with residual AHI ≥15/hr. The compliance of CT at 12 weeks and during long-term follow-up was also examined.

## 2. Materials and methods

### 2.1 Study setting

**Fig 1:**
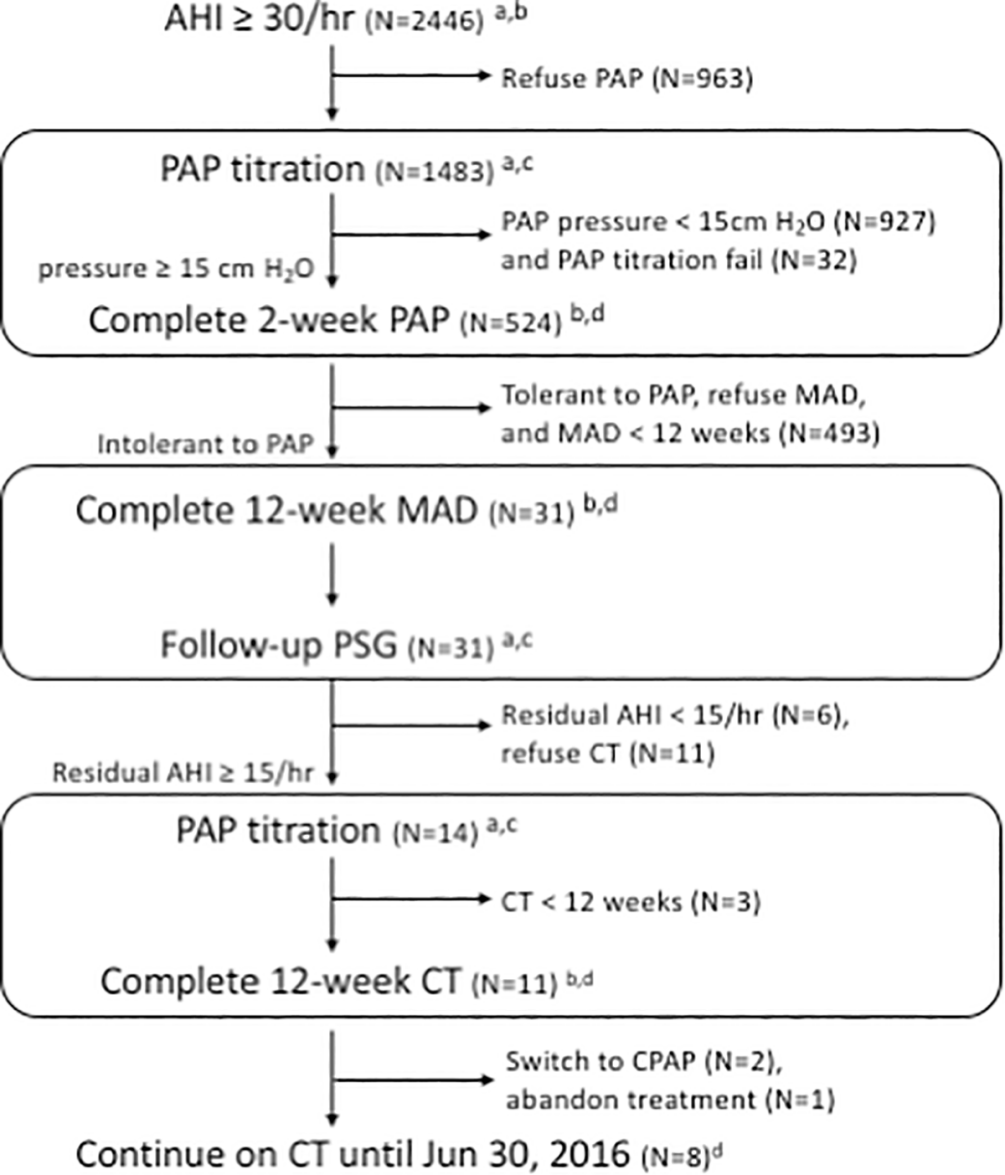
The study flow chart. Abbreviations: AHI, apnea-hypopnea index; PAP, positive airway pressure; MAD, mandibular advancement device; PSG, polysomnography; CT, combination therapy of CPAP and MAD. Letters denote data collected: a, PSG parameters; b, the Epworth Sleepiness Scale (ESS); c, PAP therapeutic pressure; d, patient compliance.

This retrospective study was conducted at the Center of Sleep Disorder of the National Taiwan University Hospital. The routine protocol for treating patients with moderate-to-severe OSA (AHI *≥*15/hr) and symptoms requiring PAP treatment was as follows (Fig 1): (1) Patients underwent a 2-week fixed-pressure PAP treatment including CPAP (S8^*™*^) or BPAP (VPAP^*™*^ IV, ResMed Inc, New South Wales, Australia) with the therapeutic pressure (P_*PAP*_) determined by overnight manual titration; (2) Patients intolerant to PAP were referred to an orthodontist for MAD; (3) Patients on MAD with residual AHI *≥*15/hr after 12-week were switched to CT of MAD and fixed–pressure CPAP with the therapeutic pressure (P_*CT*_) determined by overnight manual titration while the patient was on MAD. The patients underwent 4 repeated PSG, including pretreatment, P_*PAP*_ titration, after 12-week MAD treatment, and P_*CT*_ titration.

### 2.2 Participants

Medical records of patients diagnosed with severe OSA (AHI ≥30/hr) between October 1, 2008 and June 30, 2014 were reviewed. The inclusion criteria were: (1) Initial P_PAP_ ≥15 cm H_2_O; (2) reported intolerance to PAP because of high pressure; (3) residual AHI ≥15/hr on MAD; and (4) underwent 12 weeks of CT (Fig 1). The exclusion criterion was intolerance of PAP due to issues other than high pressure (e.q., nasal blockage). Enrolled participants who completed the 12-week CT were followed-up until June 30, 2016, with the median follow-up period of 36.5-months. Long-term compliance was obtained by reviewing medical records for patients with regular clinic visits. Patients without clinic visits were contacted by phone. The Research Ethics Committee of NTUH approved the study protocol and waived the need for consents from participants (201401086RIND).

### 2.3 Data collection

Data collected through reviewing medical records included pretreatment demographics, PSG parameters, therapeutic pressure, and morning and nocturnal BP that was recorded at the time of polysomnography. Subjective sleepiness was evaluated with the Epworth Sleepiness Scale (ESS) where excessive daytime sleepiness was defined as ESS ≥10 (Fig 1).

### 2.4 Polysomnography and measurement of overnight BP

Overnight PSG (Embla N7000, Medcare Flaga, Reykjavik, Iceland) was performed as previously reported in the sleep laboratory. The sleep stages and respiratory events were scored according to 2007 AASM scoring rule. [25] Apnea was defined as ≥90% decrease in airflow for ≥10 seconds while hypopnea was ≥30% decrease in airflow ≥10 sec associated with ≥4% reduction in arterial oxygen saturation. The PSG parameters collected included sleep efficiency, percentage of slow-wave sleep (% SWS) and % REM, AHI, oxygen desaturation index (ODI), percentage of total sleep time with SpO_2_ <90% (%TST-SpO_2_<90%), and. arousal index (AI)

Blood pressure was measured in the sitting position at 9 pm (nocturnal BP) on the night of the PSG and again at 7 am (morning BP) the next day.[26] The average of three BP measurements from each participant was used for further analysis. The overnight change in BP was calculated as the morning BP minus the nocturnal BP.

### 2.5 PAP titration and measurement of PAP compliance

The therapeutic pressure was determined by an overnight manual titration using BPAP (Synchrony^TM^, Respironics Inc., USA) following a protocol modified from the AASM recommendation.[12] For CT, the titration was done while the patient was on MAD. The choice of interface started with a nasal mask, which was changed to an oro-nasal mask if significant mouth leak was detected.

The manual titration started with the CPAP mode at pressure 4 cm H_2_O and increased by 1 cm H_2_O increments over 10 minutes to eliminate obstructive events including apnea, hypopnea, and snoring, and to minimize electroencephalography arousals until ≥30 min elapsed without obstructive event. Optimal pressure was defined as the pressure at which AHI was <5/hr for ≥15 min, including supine REM sleep.

The titration was transitioned to the BPAP mode if the obstructive events continued when CPAP pressure was 20 cm H_2_O. BPAP started at the inspiratory positive airway pressure (IPAP) of 8 cm H_2_O and the expiratory positive airway pressure (EPAP) of 4 cm H_2_O. The EPAP was increased by 1 cm H_2_O increments over 10 min to eliminate obstructive apnea. The IPAP was increased by 1 cm H_2_O for other obstructive events over ≥10 min periods and continued until ≥30 min elapsed without obstructive event. For the 14 patients underwent CT, 7 participants were prescribed CPAP and the other 7 were switched to BPAP after the first PAP titration. All 14 patients were prescribed with oro-nasal masks. At the 2^nd^ titration for CT, all 14 were prescribed with CPAP. Nasal masks were prescribed for 9 patients and oro-nasal masks were prescribed for 5 patients.

Patients starting PAP were instructed to use the device for ≥6 hr/night. Follow-up visits were scheduled at the clinic after 1, 3, 6, and 12 months during the first year, and every 6 months thereafter. PAP compliance was assessed as the average usage hours per night which was objectively recorded on the devices with a built-in compliance meter. Compliance data were downloaded by medical staff at every clinic visit.

### 2.6 Mandibular advancement device

**Fig 2:**
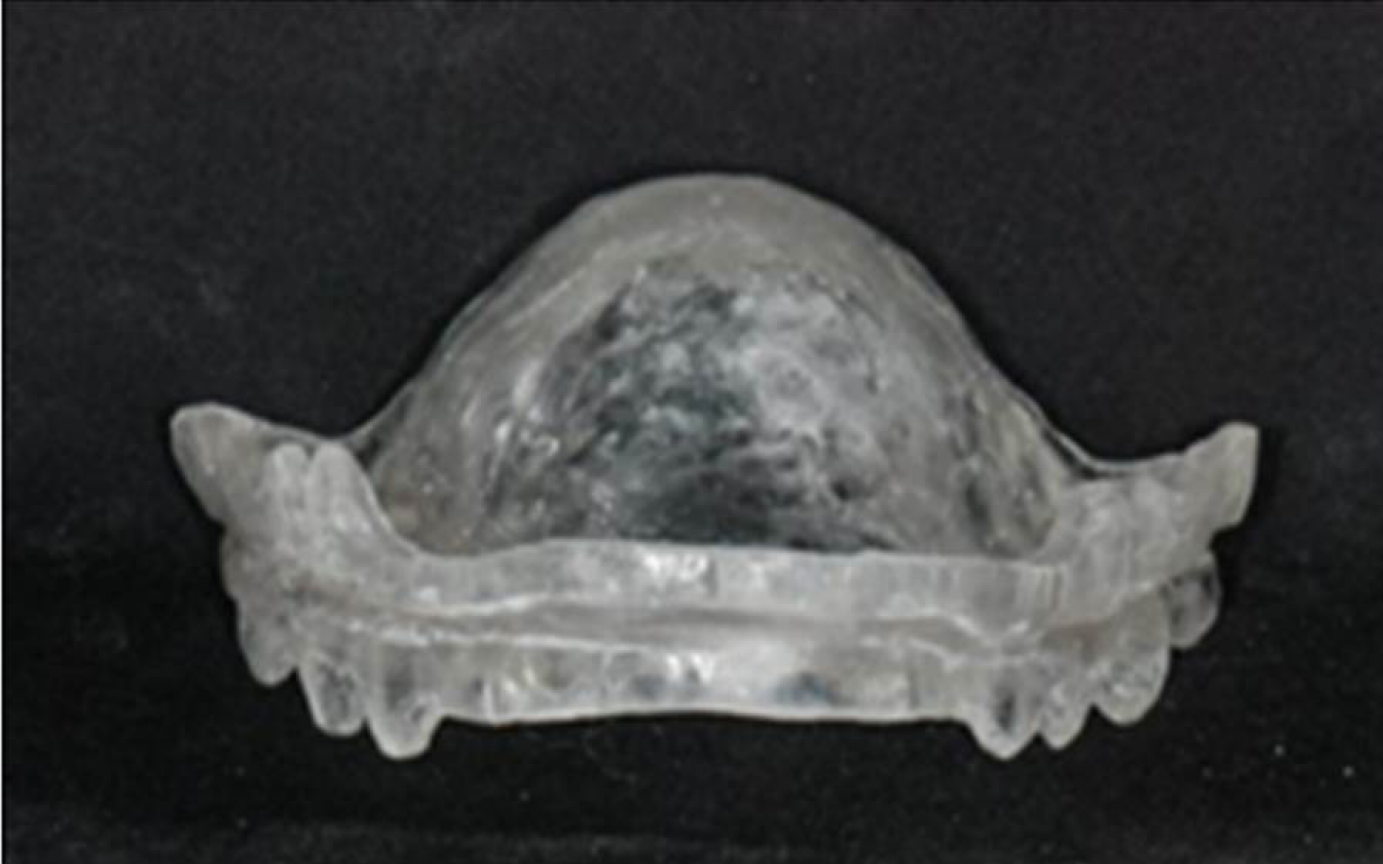
Tongue-backing mandibular advancement device (t-MAD). The device was a monobloc with resin plate designed for 75% of MaxP.

**Fig 3:**
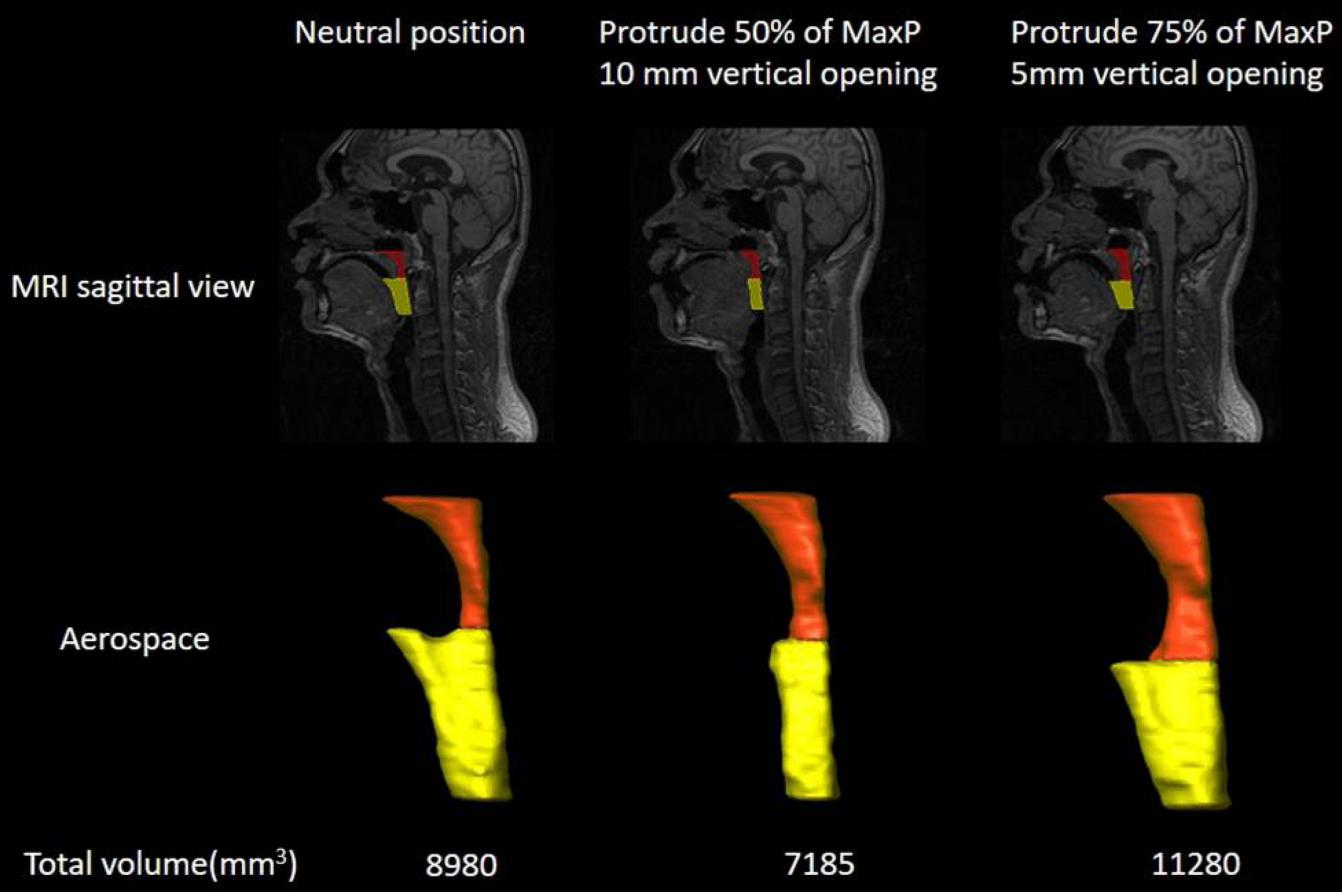
Mid-sagittal MRI demonstrating volumetric reconstruction of airway aerospace at different mandibular advancement and vertical opening in one representative patient with sleep apnea. Oral bite designed for 75% of MaxP with 5mm opening increased the total volume of upper airway more than that in neutral position or that for 50% of MaxP with 10mm opening. The retropalatal space (orange) was defined as the space between the inferior border of the hard palate and the inferior tip of the uvula. The retroglossal space (yellow) was defined as the space between the inferior border of the uvula and the superior tip of the epiglottis. The total volume is the sum of retropalatal and retroglossal volumes.

The design of MAD used in the present study was a monobloc with resin plate to support the tongue, named the tongue-backing mandibular advancement device (t-MAD) (Fig 2) [27]. The thickness and shape of the palatal coverage was designed to achieve 75% of MaxP [16, 28], while the vertical separation between the incisal edges of the maxillary and mandibular central incisors was limited to 5 mm. This design was shown to significantly increase the total volume of upper airway aerospace compared to MAD at neutral position or MAD at 50% of MaxP and 10-mm opening (Fig 3) [27]. The effect of MAD was assessed after 12 weeks, and the compliance was based on self-estimated average night usage for the entire period at each follow-up visits. The design of MAD remained the same during the study period, which allowed the comparison of the effects of MAD and CT in the cohort across the study period.

### 2.7 Outcome measurement

The primary endpoints were residual AHI, ODI, and % TST-SpO_2_ measured at PSG at pretreatment and on-treatment of PAP, MAD, and CT. The secondary endpoints were the therapeutic pressure on CT (P_CT_) compared to that on PAP (P_PAP_). The optimal P_PAP_ was recorded as 20 cm H_2_O (the maximum CPAP pressure) for patients who switched to BPAP. The tertiary endpoints were % SWS, subjective sleepiness, and overnight changes in BP. Other outcomes included sleep efficiency, % REM, and arousal index. The compliance of 2-week PAP, 12-week MAD, and 12-week and long-term CT was also recorded.

### 2.8 Statistics analysis

Continuous variables were presented as mean (standard deviation) and categorical variables as number (percentage). The treatment effects of PAP, MAD, and CT were analyzed by the Mixed linear model, with Tukey's test as the post hoc testing method to compare across the three treatment approaches. We also analyzed demographic and clinical variables that are associated with treatment response of MAD, namely the responder (residual AHI<15/hr) and non-responder (residual AHI≥15/hr). Categorical variables were tested using Fisher's exact test, while continuous variables were tested by the Mann-Whitney U test. A p-value <0.05 was considered statistically significant. All statistical analyses were performed using the SAS 9.4 software (SAS Institute, Cary, North Carolina, USA).

## 3. Results

### 3.1 Demographics

The flowchart of the study is shown in Fig 1. A total of 2446 patients with severe OSA were identified from referrals with suspected sleep apnea. Among the 1,483 patients undergoing PAP titration, PAP pressure was <15 cm H2O in 927 patients and ≥15 cm H2O in 524 patients. For the 1,451 successful titrations, CPAP was prescribed for 1,232 patients and BPAP for 219 patients. For the 524 patients with P_PAP_≥15 cm H_2_O, 493 patients were excluded due to either tolerant to PAP, refuse MAD, or MAD<12 weeks, where the majority were excluded because of tolerance to PAP. There were 31 patients intolerant to PAP who completed 12-week MAD. Only 6 patients responded to MAD treatment (responder), whereas 25 did not show significant improvement (non-responders). Compared to the responders, the non-responders had higher body mass index (BMI) (28.8±3.4 vs. 24.8±4.1 kg/m^2^, p = 0.04) and higher initial P_PAP_ (19±1.8 vs. 16.7±1.8 cm H_2_O, p = 0.02) (S1 Table).

A total of 14 patients underwent combination therapy and were included for further analysis. Pretreatment patient demographics are summarized in Table 1 and individually listed in S2 Table. Most of the 14 patients were >50 y/o (9/14), overweight or obese (12/14), and had ESS ≥10 (9/14). Six of the 14 patients had hypertension and were taking anti-hypertensive drugs. During the first PAP titration, the average therapeutic pressure 19± 1.7 cm H_2_O. The residual AHI after 12-week MAD was 43.3±19.7/hr. None of the 14 participants had MAD reset after delivery. Eleven of the 14 patients completed the 12-week combination. Side effect under CT was reported by only one patient (7.1%) who complained of dry mouth and no one reported sleep interference by CT.

**Table 1:** Pretreatment demographics of 14 patients who underwent CT.

	n = 14
Age (yr)	53.8(12.3)
BMI (kg/m^2^)	28.6(3.7)
Hypertension, n (%)	6(42.8)
Anti-hypertensive drug	6(42.8)
ESS ^a^	11.9 (4.6)
AHI (/hr)	59.2(19.8)
Nocturnal SBP	125.6(7.3)
Nocturnal DBP	76.2(9.2)
Morning SBP	127(12.2)
Morning DBP	78.4(9.8)
P_PAP_ ^b^	19 (1.7)
Residual AHI on MAD	43.3(19.7)

Data are presented as mean (standard deviation) or number (%).

Abbreviations: CT, combination therapy; BMI, body mass index; ESS, Epworth Sleepiness Scale; AHI, apnea-hypopnea index; SBP, systolic blood pressure; DBP, diastolic blood pressure; P_PAP_, therapeutic pressure of positive airway pressure; MAD, mandibular advancement device.

^a^ Data represent the 14 patients who underwent CT

^b^ For patients who transitioned to bilateral positive airway pressure (BPAP), the optimal therapeutic pressure was recorded as 20 cm H_2_O.

### 3.2 Residual AHI, ODI and % TST-SpO_2_ on combination compared to PAP or MAD

**Fig 4:**
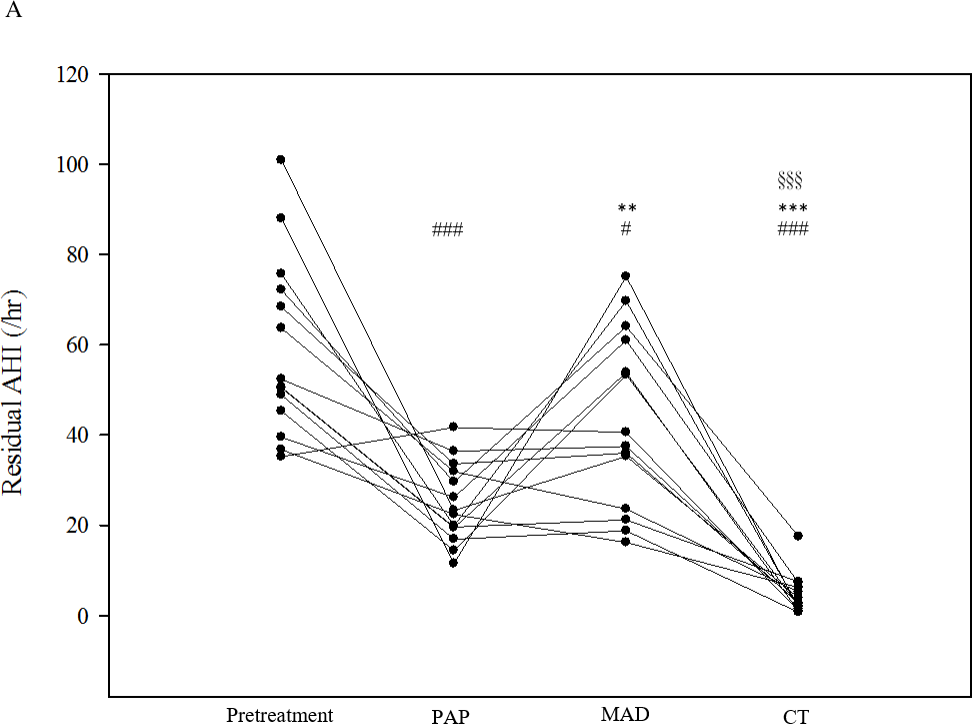
The (A) residual apnea-hypopnea index (AHI), (B) residual oxygen desaturation index (ODI), and (C) residual percentage of total sleep time with SpO_2_ <90% (%TST-SpO_2_ <90%) before and under treatments with PAP, MAD, and CT in 14 patients. PAP, continuous positive airway pressure; MAD, mandibular advancement device; CT, combination therapy. Each dot represents a measurement of an individual patient. The *p* values were analyzed by Tukey’s correction: # *p* < 0.05 and ### *p* < 0.005 compared with pretreatment values; * *p* < 0.05, ** *p* < 0.01, and *** *p* < 0.005 compared with PAP therapy; § *p* < 0.05 and §§§ *p* < 0.005 compared with MAD therapy.

BMI, polysomnographic parameters, and overnight changes in BP of 14 patients both before and after treatment with PAP, MAD, and CT are summarized in Table 2 and individually listed in the S3–S5 Tables. BMI did not change significantly and no other concurrent intervention existed across the study period. All three treatments effectively reduced AHI, ODI, and % TST-SpO_2_ <90% compared to pretreatment values. The residual AHI and ODI on CT was lower than that on MAD or PAP (Fig 4A and 4B). The residual % TST-SpO_2_ <90% on CT was lower than that on MAD and similar to that on PAP (Fig 4C).

**Table 2:** Body mass index, polysomnographic parameters, and overnight changes in blood pressure before and during treatments with PAP, MAD, and CT in the 14 patients who underwent CT.

	Pretreatment	PAP	MAD	CT	*p* ^a^
BMI (kg/m^2^)	28.6(3.7)	28.6(3.5)	28.6(3.2)	28.4(3.4)	0.71
ESS ^b^	11.5(4.3)	9.8(2.2)	9.6(3.5)	7.9(4)	0.01
Sleep efficiency (%)	79.7(12)	77.8(10)	78.9(14.8)	84.7(10.1)	0.42
SWS (%)	0(0.1)	1.5(3.5)	2.4(4.7)	3.1(7.9)	0.38
REM (%)	14.5(7.4)	14.9(8.5)	18.2(6.9)	21(9.4)	0.07
AHI (/hr)	59.2(19.8)	24.8(8.8)	43.3(19.7)	4.4(4.4)	<0.001
ODI (/hr)	58.4(20.5)	20.3(9)	43.3(18.9)	5.6(5.9)	<0.001
% TST-SpO_2_ <90% (%)	22.9(13.5)	2.1(2.8)	9.7(9.4)	0.3(0.5)	<0.001
Arousal index (%)	24.3(16.2)	15.9(7.4)	19.4(10.3)	6.1(3.5)	<0.001
Overnight change in SBP	1.4(11)	-3.5(8.7)	4.8(10.5)	2.8(11.9)	0.24
Overnight change in DBP	2.2(5.2)	-3(8.6)	4.5(8.2)	-0.2(7.0)	0.06

Data are presented as mean (standard deviation).

^a^ *p* values were analyzed by the mixed effect model.

^b^ Data only considered the 11 patients who completed 12 weeks of CT.

Abbreviations: PAP, continuous positive airway pressure; MAD, mandibular advancement device; CT, combination therapy; BMI, body mass index; ESS, Epworth Sleepiness Scale; AHI, apnea-hypopnea index; ODI, oxygen desaturation index; % TST-SpO_2_ <90%, % total sleep time with oxygen saturation <90%; SWS, slow-wave sleep; SBP, systolic blood pressure; DBP, diastolic blood pressure.

### 3.3 Therapeutic pressure on CT compared with PAP

**Fig 5:**
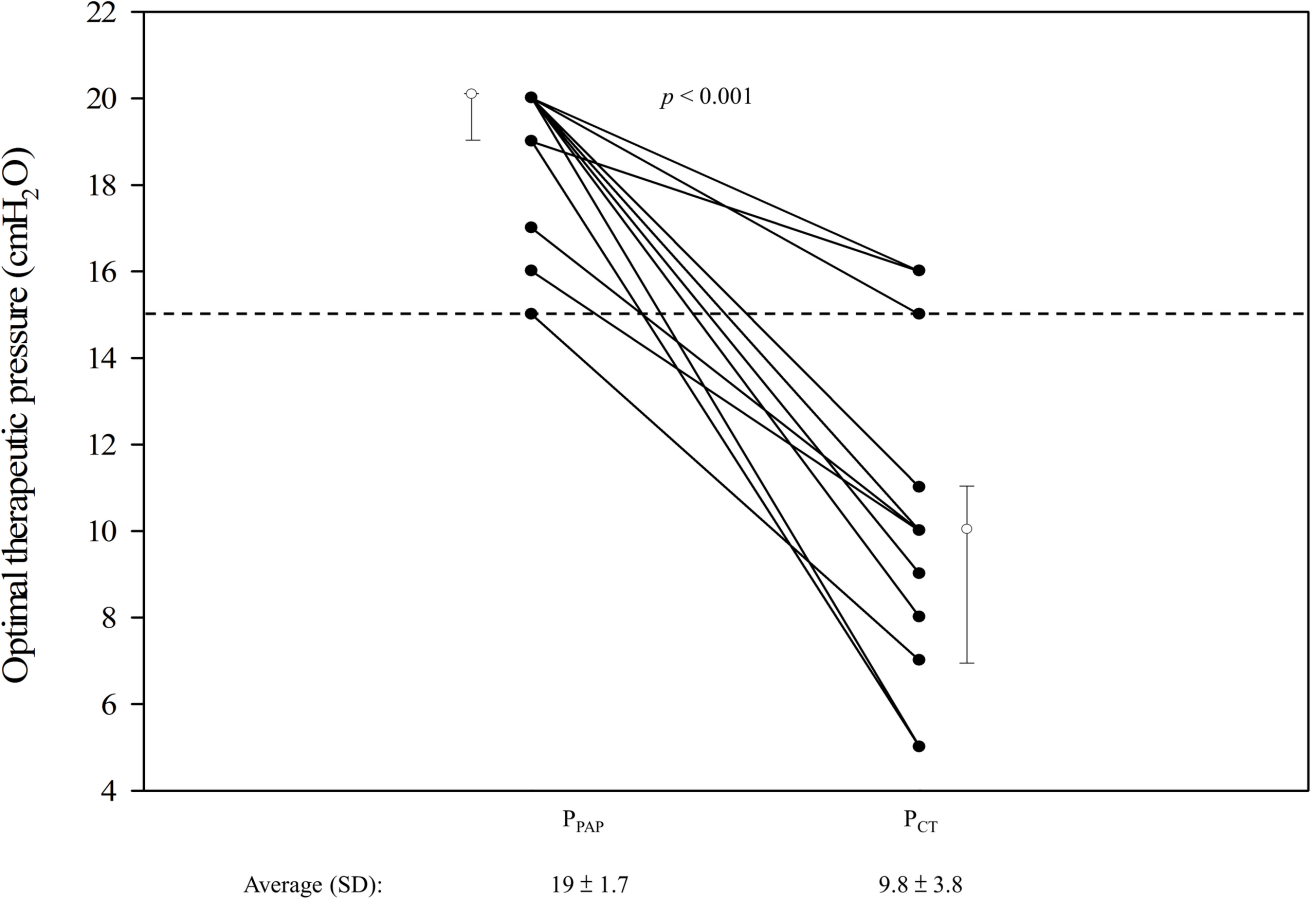
Optimal therapeutic pressure of PAP (P_PAP_) and CT (P_CT_) in 14 patients. The figure shows the individual pressure (black dots), the median (white dots), and the 25% and 75% quantiles (whiskers) on PAP and CT. PAP, continuous positive airway pressure; CT, combination therapy. Each solid line connects data of an individual patient. Dash line indicates 15 cm H_2_O.

The average therapeutic pressure on CT was 9.8±3.8 cm H_2_O, which was significantly lower than that on PAP (19±1.7 cm H_2_O, p<0.001) (Fig 5). Although all patients had lowered P_CT_ than P_PAP_, the therapeutic pressure remained ≥15 cm H_2_O in 3 patients (15 cm H_2_O in one and 16 cm H_2_O in two).

### 3.4 Changes in % SWS, ESS, and overnight changes in blood pressure when patients were on PAP, MAD, and CT

For the 11 patients who completed 12 weeks of CT, only CT reduced ESS compared to that before treatment (7.9±4 vs 11.5±4.3, *p* = 0.008). However, the average residual ESS after CT (7.9±4) was not statistically different from that after PAP (9.8±2.2, *p* = 0.26) or MAD (9.6±3.5, *p* = 0.34) (Table 2).

In the 14 patients, none of the three treatments had significant impact on % SWS and overnight changes of BP, sleep efficieny, and %REM. The residual arousal index on CT was lower than that at pretreatment (p<0.001) or MAD (p = 0.006), and was similar to that on PAP (p = 0.061).

### 3.5 Compliance of PAP, MAD, and CT

In 14 patients, the average usage of the 2-week PAP and the 12-week MAD was 2.9±1.1 hr/night and 6.4±1.1 hr/night, respectively. For the 11 patients who underwent CT for at least 12 weeks, the average usage at the 12th week was 5.9±1.7 hr/night. During a median follow-up of 36.5-months, eight patients (72.7%) remained on CT with an average usage of 6.4±1.5 hr/night, while two patients switched to CPAP because of dental problems and one patient abandoned all treatment.

## 4. Discussion

The present study found that combining MAD and PAP showed additive effect for treating patients with severe OSA who were intolerant to PAP ≥15 cm H_2_O and had residual AHI ≥15/hr under MAD. The participants of the present study were limited to patients who had severe OSA severity including high pretreatment AHI, high P_PAP_, and high residual AHI on MAD. Although all three treatments effectively reduced AHI, ODI, and % TST-SpO_2_ <90% compared to pretreatment values, the residual AHI and ODI on CT was lower than those on MAD or PAP, and the residual % TST-SpO_2_ <90% was lower than that on MAD. The result also showed that only CT significantly reduced ESS compared to baseline. In addition, CT significantly reduced the optimal treatment pressure (P_CT_) compared with PAP alone (P_PAP_). For patients remained on CT during the follow-up period, the average nightly usage during follow-up (6.4±1.5 hr/night) was comparable to that at the 12^th^ week of CT (5.9±1.7 hr/night), indicating good long-term compliance.

Unlike the titratable MAD used in previous studies by El-Solh *et al*. and de Vries *et al*. [22, 23], the present study used MAD of a monoblock designed for 75% of maximum mandibular advancement. Previous studies has shown that monoblocks had non-inferior effects compared with titratable MAD [29]. The design of 75% of MaxP with 5-mm vertical opening was based on the findings of previous studies: MAD at 75% of MaxP was shown to achieve higher success rate than those at 50% of MaxP in patients with severe OSA [16], and increased vertical mouth opening adversely affect the upper airway patency in sleep apnea patients [28]. The MRI scanning of upper airway aerospace also showed 75% of MaxP and a 5-mm opening significantly increased the total volume of pharyngeal aerospace compared to either neutral position or 50% of MaxP and 10-mm opening (Fig 4). Although advancement over 50% of MaxP has been associated with increase in side effect for two-piece MAD, the only reported side effect in our study was dry mouth in one patient.

The present study showed that the residual AHI and ODI on CT was lower than that on MAD or PAP, which was different from that in the findings reported by El-Solh *et al*. and de Vries *et al*., both reporting that CT and CPAP had similar effects on reducing AHI [22, 23]. Moreover, we showed that only CT reduced ESS compared to the pretreatment value, also in contrasted with the El-Solh’s study where both MAD and combination reduced ESS. [22] The discrepancies between the present study and El-Solh’s study are likely due to differences in patient characters, *i*.*e*., the present study had more male (100% vs 40%) and patients with more severe OSA (mean AHI 59.2 vs. 23.5/hr). Though the sex ratio and OSA severity were similar between present study and de Vries’s study, the present study recruited patients intolerant to PAP therapy where the 5 participants in de Vries’s study were well-tolerated to CPAP before being fitted for CT.

We showed that the therapeutic pressure was lowered by an average of 9.2 cm H_2_O on CT compared with PAP. Such a reduction was much more than what was reported by El-Solh *et al*. (2 cm H_2_O) and de Vries *et al*. (5.1 cm H_2_O), possibly due to the higher initial P_PAP_ (19±1.7) in the present study than that in El-Solh’s (9.4±2.3) and de Vries’s (11.5±1.3) studies. Kaminska *et al*. [24, 30] reported no effect on therapeutic pressure with MAD set for neutral position, suggesting that the effect may be related to improved airflow at 75% of MaxP. As a result, such a significant lowering of therapeutic pressure may have contributed to good long-term compliance.

The present study has several limitations. First, this is a retrospective study with a highly-selected, small cohort; thus, the findings may not be applicable for patients with different OSA characteristics. Also, the small case number and thus modest statistical power of this study may lead to the non-significant results in ESS, %SWS, and BP between CT and the other treatments. Second, the P_PAP_ of patients who received BPAP was recorded as 20 cm H_2_O, which is the maximum pressure of CPAP. This may introduce a bias that underestimate the initial P_PAP_ and thus undermine the effect of CT on therapeutic pressure, although our results already show that CT greatly reduced therapeutic pressure. Third, there was no wash-out period between MAD and CT, which may in part contribute to the effect of CT. Nonetheless, the present study showed a clear additive effect of CT on AHI and ODI, providing the basis for further investigation. A prospective, large-scale study is warranted to validate the aforementioned findings.

In conclusion, results of the present study support that combining CPAP and MAD may have additive effect on reducing apnea-hypopnea events, oxygen desaturation, sleepiness, and reduced therapeutic pressure.

## Supporting information

S1 Table Comparison of pretreatment and P_PAP_ of patients with MAD responder and non-responder. (PDF)

S2 Table Pretreatment demographics and P_PAP_ of 14 patients who underwent CT. (PDF)

S3 Table Apnea-hypopnea index (AHI) before and under treatment for the 14 patients who underwent CT. (PDF)

S4 Table Oxygen desaturation index (ODI) before and under treatment for the 14 patients who underwent CT. (PDF)

S5 Table % total sleep time (TST) of SpO_2_ <90% before and under treatment for the 14 patients who underwent CT. (PDF)

## Abbreviations

AHI: apnea-hypopnea index
BMI: body mass index
BP: blood pressure
BPAP: bilevel positive airway pressure
CPAP: continuous positive airway pressure
DBP: diastolic blood pressure
ESS: Epworth Sleepiness Scale
EPAP: expiratory positive airway pressure
hr: hour
IPAP: inspiratory positive airway pressure
MaxP: maximum protrusion
MAD: mandibular advancement devices
ODI: oxygen desaturation index
OSA: obstructive sleep apnea
PAP: positive airway pressure
PSG: polysomnography
SBP: systolic blood pressure
SD: standard deviation
SpO_2_: peripheral capillary oxygen saturation
TST: total sleep time
