# Correction: Combining MAD and CPAP as an effective strategy for treating patients with severe sleep apnea intolerant to high-pressure PAP and unresponsive to MAD

**DOI:** 10.1371/journal.pone.0196319

**Published:** 2018-04-19

**Authors:** Hsiang-Wen Liu, Yunn-Jy Chen, Yi-Chun Lai, Ching-Yi Huang, Ya-Ling Huang, Ming-Tzer Lin, Sung-Ying Han, Chi-Ling Chen, Chong-Jen Yu, Pei-Lin Lee

Fig 4 is incorrect. Please see the corrected Fig 4 here.

**Fig 4:**
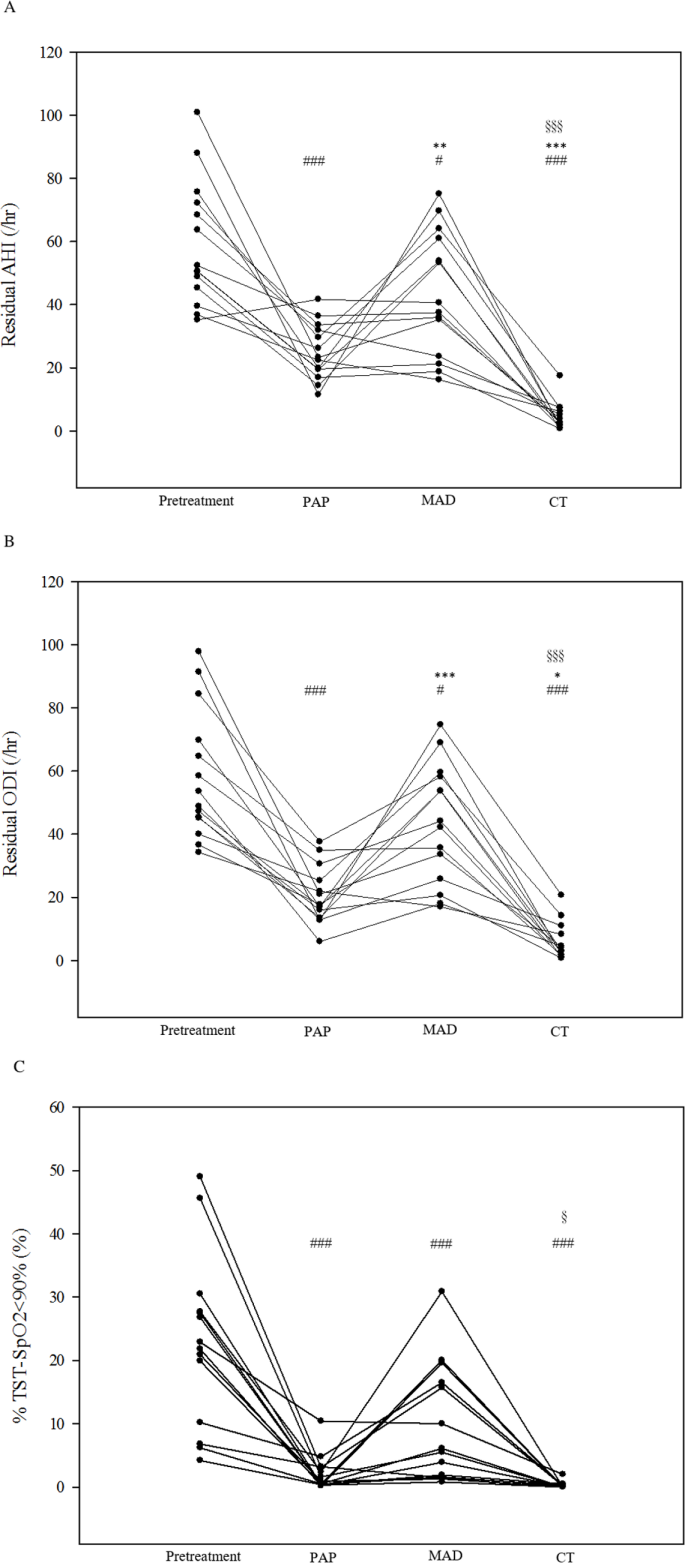
The (A) residual apnea-hypopnea index (AHI), (B) residual oxygen desaturation index (ODI), and (C) residual percentage of total sleep time with SpO_2_ <90% (%TST-SpO_2_ <90%) before and under treatments with PAP, MAD, and CT in 14 patients. PAP, continuous positive airway pressure; MAD, mandibular advancement device; CT, combination therapy. Each dot represents a measurement of an individual patient. The *p* values were analyzed by Tukey’s correction: # *p* < 0.05 and ### *p* < 0.005 compared with pretreatment values; * *p* < 0.05, ** *p* < 0.01, and *** *p* < 0.005 compared with PAP therapy; § *p* < 0.05 and §§§ *p* < 0.005 compared with MAD therapy.
