# Supplementary material for: Combining MAD and CPAP as an effective strategy for treating patients with severe sleep apnea intolerant to high-pressure PAP and unresponsive to MAD
Source: PLoS One. 2017 Oct 26;12(10):e0187032. doi: 10.1371/journal.pone.0187032 (PMC5658160; doi:10.1371/journal.pone.0187032)
Supplement: S1 Table — (PDF) [file pone.0187032.s001.pdf]

Table S1. Comparison of pretreatment and P_{PAP} of patients with MAD responder and non-responder

	responder (n=6)	non-responder (n=25)	*p*
Age (yr)	56(5.4)	54(13.8)	0.76
Male (%)	50	80.1	0.11
BMI (kg/m^2)	24.8(4.1)	28.8(3.4)	0.04
CAD (%)	16.7	12	1
Hypertension (%)	16.7	60	0.08
Anti-HT drug (%)	16.7	56	0.09
ESS	12.7(2.6)	11.2(4.1)	0.31`
AHI (/hr)	45.9(12.4)	62.3(22.1)	0.1
P_{PAP}^{*}(cmH_2O)	16.7(1.8)	19.0(1.8)	0.02

Data presented as mean (SD) or number (%)

Abbreviations: MAD, mandibular advancement device; BMI, body mass index; CAD, coronary artery disease; HT, hypertension; ESS, Epworth Sleepiness Scale; AHI, apnea-hypopnea index; P_{PAP}, optimal pressure of positive airway pressure therapy
