# Supplementary material for: Combining MAD and CPAP as an effective strategy for treating patients with severe sleep apnea intolerant to high-pressure PAP and unresponsive to MAD
Source: PLoS One. 2017 Oct 26;12(10):e0187032. doi: 10.1371/journal.pone.0187032 (PMC5658160; doi:10.1371/journal.pone.0187032)
Supplement: S2 Table — (PDF) [file pone.0187032.s002.pdf]

Table S2. Pretreatment demographics and P_{PAP} of 14 patients who underwent CT

	Age (yr)	BMI (kg/m^2)	Co-morbidity	anti-hypertensive drug	ESS	AHI (/hr)	P_{PAP} (cm H_2O) [a]
Case 1	64	31.4	HT/CAD	Yes	20	68.4	20
Case 2	56	26.5	HT	Yes	8	48.9	16
Case 3	43	35.1	HT	Yes	9	75.7	20
Case 4	42	23.2	No	No	16	63.7	20
Case 5	62	28.8	No	No	14	45.3	19
Case 6	43	34.8	No	No	11	88	19
Case 7	73	28.9	HT	Yes	12	35.2	20
Case 8	34	29.6	No	No	13	39.5	17
Case 9	56	27.6	HT	Yes	12	50.6	20
Case 10	54	30.9	No	No	9	100.9	15
Case 11	35	29.5	No	No	6	72.2	20
Case 12	69	26.4	HT/CAD	Yes	13	50.5	20
Case 13	59	22.7	No	No	7	36.8	20
Case 14	62	25.1	No	No	20	52.4	20

Abbreviations: CT, combination therapy; BMI, body mass index; ESS, Epworth Sleepiness Scale; AHI, apnea-hypopnea index; P_{PAP}, optimal pressure of positive airway pressure therapy; HT, hypertension; CAD, coronary artery disease. [a]: For patients who transitioned to bilateral positive airway pressure (BPAP), the optimal therapeutic pressure was recorded as 20 cm H_2O.
