# Supplementary material for: Combining MAD and CPAP as an effective strategy for treating patients with severe sleep apnea intolerant to high-pressure PAP and unresponsive to MAD
Source: PLoS One. 2017 Oct 26;12(10):e0187032. doi: 10.1371/journal.pone.0187032 (PMC5658160; doi:10.1371/journal.pone.0187032)
Supplement: S3 Table — (PDF) [file pone.0187032.s003.pdf]

Table S3. Apnea-hypopnea index (AHI) before and under treatment for the 14 patients who underwent CT

	Pretreatment (/hr)	PAP (/hr)	MAD (/hr)	CT (/hr)
Case 1	68.4	33.6	36	2.8
Case 2	48.9	16.9	18.8	0.7
Case 3	75.7	19.9	69.7	1.9
Case 4	63.7	31.9	23.6	5.2
Case 5	45.3	14.4	53.4	2.1
Case 6	88	11.5	75.1	1.4
Case 7	35.2	41.7	40.6	1
Case 8	39.5	26.2	61	7.2
Case 9	50.6	19.6	21.2	7.5
Case 10	100.9	23.3	35.3	3.9
Case 11	72.2	29.6	64.1	17.5
Case 12	50.5	19.5	53.9	0.9
Case 13	36.8	22.4	16.2	6.2
Case 14	52.4	36.4	37.5	2.6
Mean	59.2	24.8	43.3	4.4
SD	19.4	8.8	19.7	4.4

Abbreviations: PAP, positive airway pressure; MAD, mandibular advancement device; CT, combination therapy; SD, standard deviation
