# Supplementary material for: Combining MAD and CPAP as an effective strategy for treating patients with severe sleep apnea intolerant to high-pressure PAP and unresponsive to MAD
Source: PLoS One. 2017 Oct 26;12(10):e0187032. doi: 10.1371/journal.pone.0187032 (PMC5658160; doi:10.1371/journal.pone.0187032)
Supplement: S4 Table — (PDF) [file pone.0187032.s004.pdf]

Table S4. Oxygen desaturation index (ODI) before and under treatment for the 14 patients who underwent CT

	Pretreatment	PAP	MAD	CT
Case 1	64.7	34.9	35.7	1.7
Case 2	45.2	16	20.6	0.7
Case 3	69.8	17	69	1.8
Case 4	53.6	6	18	4.6
Case 5	45.4	13.5	53.8	2.7
Case 6	91.4	12.8	74.7	20.7
Case 7	36.6	17.7	42.2	1.2
Case 8	40	25.3	59.6	3.1
Case 9	48.8	12.9	25.8	11
Case 10	97.8	21	33.6	4.2
Case 11	84.4	37.6	58.2	14.2
Case 12	47.3	17.2	53.7	1
Case 13	34.2	21.9	17	8.3
Case 14	58.5	30.6	44.2	2.7
Mean	58.4	20.3	43.3	5.6
SD	20.5	9	18.9	5.9

Abbreviations: PAP, positive airway pressure; MAD, mandibular advancement device; CT, combination therapy; SD, standard deviation
