# Supplementary material for: Combining MAD and CPAP as an effective strategy for treating patients with severe sleep apnea intolerant to high-pressure PAP and unresponsive to MAD
Source: PLoS One. 2017 Oct 26;12(10):e0187032. doi: 10.1371/journal.pone.0187032 (PMC5658160; doi:10.1371/journal.pone.0187032)
Supplement: S5 Table — (PDF) [file pone.0187032.s005.pdf]

Table S5. % total sleep time (TST) of SpO_2 <90% before and under treatment for the 14 patients who underwent CT

	Pretreatment	PAP	MAD	CT
Case 1	6.8	3.2	1.5	0.3
Case 2	26.8	0.3	0.8	0
Case 3	27.7	2.4	30.9	0
Case 4	4.2	0.4	1.6	0.2
Case 5	19.9	0.5	6.1	0
Case 6	27.5	0.3	3.9	0.1
Case 7	21.8	0.2	19.6	0
Case 8	10.2	4.8	16.5	0.3
Case 9	20.9	0.9	1.3	0
Case 10	30.5	0.4	1.9	0.5
Case 11	49	3.1	15.7	0.2
Case 12	6.2	0.5	20	0
Case 13	22.9	10.4	10	2
Case 14	45.6	1.6	5.5	0
Mean	22.9	2.1	9.7	0.3
SD	13.5	2.8	9.4	0.5

Abbreviations: PAP, positive airway pressure; MAD, mandibular advancement device; CT, combination therapy; SD, standard deviation
